# Soil Functional Zone Management: A Vehicle for Enhancing Production and Soil Ecosystem Services in Row-Crop Agroecosystems

**DOI:** 10.3389/fpls.2016.00065

**Published:** 2016-02-05

**Authors:** Alwyn Williams, Daniel A. Kane, Patrick M. Ewing, Lesley W. Atwood, Andrea Jilling, Meng Li, Yi Lou, Adam S. Davis, A. Stuart Grandy, Sheri C. Huerd, Mitchell C. Hunter, Roger T. Koide, David A. Mortensen, Richard G. Smith, Sieglinde S. Snapp, Kurt A. Spokas, Anthony C. Yannarell, Nicholas R. Jordan

**Affiliations:** ^1^Department of Agronomy and Plant Genetics, University of Minnesota, St PaulMN, USA; ^2^Department of Plant, Soil and Microbial Sciences, Michigan State University, East LansingMI, USA; ^3^Department of Natural Resources and the Environment, University of New Hampshire, DurhamNH, USA; ^4^Department of Natural Resources and Environmental Sciences, University of Illinois at Urbana–Champaign, UrbanaIL, USA; ^5^Global Change and Photosynthesis Research Unit, United States Department of Agriculture – Agricultural Research Service, UrbanaIL, USA; ^6^Department of Plant Science, The Pennsylvania State University, University ParkPA, USA; ^7^Department of Biology, Brigham Young University, ProvoUT, USA; ^8^Soil and Water Management Unit, United States Department of Agriculture – Agricultural Research Service, St PaulMN, USA

**Keywords:** crop yield, ecosystem services, precision tillage, soil biodiversity, soil management, temporal intensification, trade-offs, zonal tillage

## Abstract

There is increasing global demand for food, bioenergy feedstocks and a wide variety of bio-based products. In response, agriculture has advanced production, but is increasingly depleting soil regulating and supporting ecosystem services. New production systems have emerged, such as no-tillage, that can enhance soil services but may limit yields. Moving forward, agricultural systems must reduce trade-offs between production and soil services. Soil functional zone management (SFZM) is a novel strategy for developing sustainable production systems that attempts to integrate the benefits of conventional, intensive agriculture, and no-tillage. SFZM creates distinct functional zones within crop row and inter-row spaces. By incorporating decimeter-scale spatial and temporal heterogeneity, SFZM attempts to foster greater soil biodiversity and integrate complementary soil processes at the sub-field level. Such integration maximizes soil services by creating zones of ‘active turnover’, optimized for crop growth and yield (provisioning services); and adjacent zones of ‘soil building’, that promote soil structure development, carbon storage, and moisture regulation (regulating and supporting services). These zones allow SFZM to secure existing agricultural productivity while avoiding or minimizing trade-offs with soil ecosystem services. Moreover, the specific properties of SFZM may enable sustainable increases in provisioning services via temporal intensification (expanding the portion of the year during which harvestable crops are grown). We present a conceptual model of ‘virtuous cycles’, illustrating how increases in crop yields within SFZM systems could create self-reinforcing feedback processes with desirable effects, including mitigation of trade-offs between yield maximization and soil ecosystem services. Through the creation of functionally distinct but interacting zones, SFZM may provide a vehicle for optimizing the delivery of multiple goods and services in agricultural systems, allowing sustainable temporal intensification while protecting and enhancing soil functioning.

## Introduction

Intensification of agriculture has been vital for increasing global food supply and alleviating hunger for millions of people (Godfray et al., 2010). In addition, intensification is key to meeting growing demand for bioenergy feedstocks and a wide variety of bio-based products (Jordan et al., 2007; McCormick and Kautto, 2013). However, agricultural intensification has also resulted in damage to the environment. In particular, soils in many regions of the world have been degraded by intensive agricultural practices (Mäder et al., 2002; Tilman et al., 2002; Heenan et al., 2004), and this has led to increased societal demand for more sustainable agricultural production systems (Foley et al., 2011; Kremen and Miles, 2012). In response, new management strategies have emerged, including soil-focused approaches such as no-tillage, which aim to improve soil regulating and supporting ecosystem services by reducing soil disturbance (Hobbs et al., 2008; Baveye et al., 2011; Palm et al., 2014). However, no-tillage often results in reduced yields (Giller et al., 2009; Pittelkow et al., 2015), highlighting trade-offs between soil and provisioning services. Such trade-offs are highly problematic, given that global demand for food and other agricultural products is expected to rise considerably by 2050 (Godfray et al., 2010; Tilman et al., 2011). Furthermore, to limit the need to convert additional lands to agriculture (i.e., extensification), the world’s existing crop production systems must become more productive (Foley et al., 2011; Bommarco et al., 2013; Godfray and Garnett, 2014).

One option for securing the productivity of existing agricultural land while also enhancing delivery of soil ecosystem services is by integrating the high productivity of intensive field crop production systems (including intensive tillage) with the improvements in soil quality associated with stringent limitations on tillage. Herein, we present evidence that a novel approach to management of field crop agroecosystems – soil functional zone management (SFZM) – can promote such integration. As detailed below, SFZM entails the creation and management of distinct yet complementary soil functional zones that have potential to reduce trade-offs between short-term productivity and soil quality.

We believe SFZM to be a previously unrecognized strategy for expanding the range of ecosystem service production from field crop agroecosystems. Several forms of SFZM (e.g., ridge tillage and strip tillage) have been studied extensively in terms of their effects on a range of crop and soil attributes. Here, we expand upon this level of analysis and understanding through a broad exploration of ecosystem service production and underlying agroecological processes in SFZM, drawing on a wide range of evidence and identifying critical knowledge gaps in understanding of SFZM. In our analysis, we focus first on supporting and regulating services, and then examine the potential of SFZM to increase productivity of agricultural systems (i.e., enhance provisioning services). In particular, we consider the role of SFZM in supporting temporal intensification, which aims to enhance provisioning services by expanding the annual time period in which harvestable crops are grown. We consider the potential dynamics of agroecosystems under SFZM, and the role of these dynamics in improving the sustainability of temporal intensification. We focus on the dynamic implications of ‘virtuous cycles’ (self-reinforcing feedback processes with desirable effects) that may occur in SFZM. Such feedback processes may serve to reduce trade-offs between provisioning, supporting, and regulating services in temporal intensification.

## Soil Functional Zone Management

**FIGURE 1:**
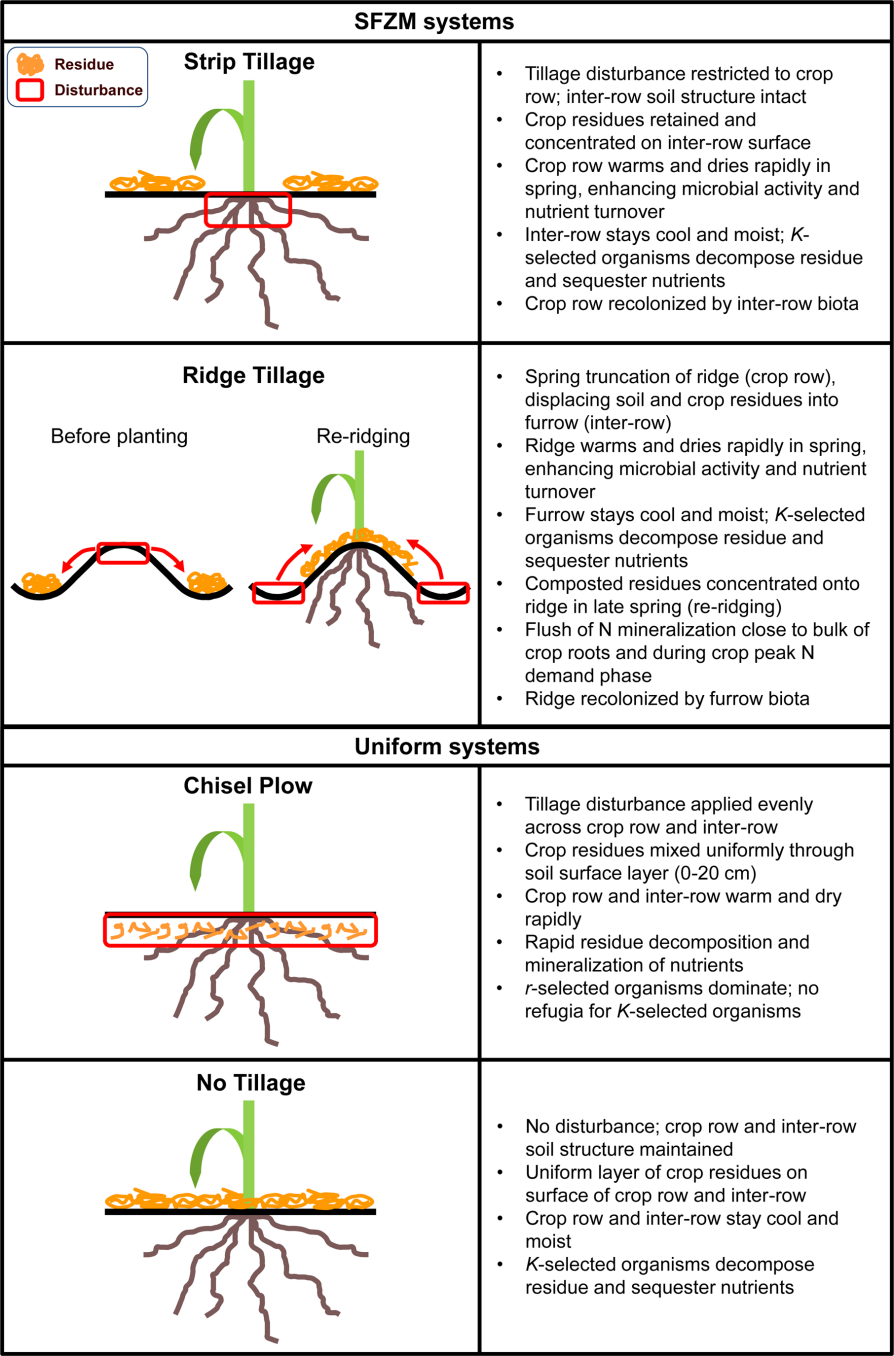
**Examples of typical soil functional zone management (SFZM) and uniform tillage systems**.

Soil functional zone management is a novel concept of field crop agroecosystem management that seeks to create distinct, yet functionally complementary soil zones through non-uniform management of tillage and crop residues. These zones can be tailored for a variety of different functions or ecosystem services and can be permanent or change locations between seasons. At its most basic, SFZM involves a zone of ‘active turnover’, managed to optimize conditions for seed germination and crop growth; and an adjacent ‘soil building’ zone, which is managed to protect soil organic matter (SOM), enhance soil water holding capacity and provide habitats for soil organisms. At present, the two most widely practiced implementations of SFZM are ridge tillage and strip tillage (**Figure 1**). While SFZM does not necessarily involve novel management practices (e.g., ridge tillage has been practiced since the 1980s), it provides a novel framework for enhancing ecosystem service production in field crop agroecosystems.

Soil functional zone management differs markedly from conventional and no-tillage practices, which can both be characterized as non-zonal, or uniform. For example, in a chisel plow system, topsoil and crop residues are uniformly mixed, creating a relatively homogenous soil environment across a tilled field (Mannering and Fenster, 1983). In no-tillage, the soil is left undisturbed and crop residues are retained, providing uniform residue cover on the soil surface (Mannering and Fenster, 1983; Hobbs et al., 2008; **Figure 1**). Despite advances in precision agricultural application of fertilizer and agrochemicals, tillage is still predominantly applied homogeneously (Lal, 2015).

Through creation and management of differentiated soil zones (**Figure 1**), SFZM creates spatial heterogeneity over small (<1 m) spatial scales. Relative to non-zonal tillage, such enhancement of within-field heterogeneity across space and time serves to enhance the range of soil physical conditions and functional biodiversity within a row-crop agroecosystem. Increasing heterogeneity can enhance biodiversity by providing habitat and other key resources to a wider range of organisms. This expansion of resource diversity in space and time can support effective resource partitioning and increased diversity of microhabitats, allowing coexistence of soil organisms and increased functional biodiversity (Ettema and Wardle, 2002; Kremen and Miles, 2012). In turn, increased functional biodiversity can support provisioning services while simultaneously conserving or enhancing a range of soil services, including organic matter decomposition and nutrient turnover, soil carbon storage, and pathogen suppression (Coleman et al., 2004; Birkhofer et al., 2008; van der Heijden et al., 2008; Suzuki et al., 2013).

Conventional soil management is typically characterized by frequent and intense disturbance (e.g., tillage and agrochemicals) combined with low plant resource diversity (e.g., monocultures and minimal crop residue). These factors lead to reduced abundances and diversity of soil organisms in conventional systems compared with no-tillage and other systems with reduced tillage and more diverse crop rotations (Wardle, 1995; Kabir, 2005; Culman et al., 2010; Postma-Blaauw et al., 2010). Moreover, these factors selectively alter soil biotic communities, leading to dominance by *r*-selected organisms (organisms adapted for rapid reproduction and dispersal; Pianka, 1970; Verbruggen and Kiers, 2010). For example, larger-bodied soil organisms are reduced in abundance relative to smaller-bodied organisms, leading to reductions in faunal and fungal biomass, and shifts toward bacterial dominance (de Vries et al., 2006; Postma-Blaauw et al., 2010). The adoption of no-tillage management has been demonstrated to improve the abundance and diversity of soil communities, such that they more closely resemble undisturbed grasslands (Postma-Blaauw et al., 2012; Säle et al., 2015). SFZM entails limited and targeted disturbance across both space and time, and maintenance of crop residues, thereby providing undisturbed or minimally disturbed soil refugia (**Figure 1**). We hypothesize that these refugia can support faunal and fungal diversity in a similar way to no-tillage, and provide a base from which slow-growing organisms with longer generation times (*K*-selected organisms) might be able to recolonize disturbed areas. In essence, we propose that SFZM, by expanding both habitat and resources relative to conventional soil management, can enhance both provisioning and soil regulating and supporting services by enhancing soil biodiversity.

## SFZM and Soil Ecosystem Services

Securing high levels of agricultural production while simultaneously improving regulating and supporting soil ecosystem services requires management strategies that expand the range of service production (Foley et al., 2011; Bommarco et al., 2013). As outlined above, by providing spatial and temporal heterogeneity in terms of tillage and crop residue distribution, we hypothesize that SFZM is one such strategy. In the following sections, we present and examine evidence that SFZM can, in fact, enhance soil ecosystem service delivery.

### Supporting Soil Services

#### Services Produced by Soil Biota

The creation of undisturbed refugia for soil microbiota, particularly filamentous fungi, through targeted disturbance is one pathway by which SFZM may increase the supply of supporting soil services. Such refugia should impact carbon (C), nitrogen (N), and phosphorus (P) cycling to the benefit of above-ground productivity. Nutrient cycling among organic and inorganic pools is driven by microbial turnover, with fungi generally thought to be more effective at storing C and N in organic matter than bacteria (Six et al., 2006), while the higher turnover rate of bacteria promotes gross mineralization and plant nutrient uptake (Schimel and Bennett, 2004). Filamentous, saprophytic fungi are also the dominant decomposers of recalcitrant plant litter, producing more degradative enzymes than bacteria (Treseder and Lennon, 2015). Arbuscular mycorrhizal fungi (AMF), meanwhile, are well-known to dominate plant P nutrition, and their central role in C and N cycling is increasingly recognized (Hodge and Storer, 2015). Thus, a combination of bacteria- and fungi-rich communities is desirable for efficient nutrient cycling.

Soil communities under conventional tillage generally have altered structural, morphological, and functional profiles compared to communities under no-tillage. Overall, tillage lowers microbial biomass, enzyme activities, and nutrient cycling rates (Kladivko, 2001; Balota et al., 2014). While tillage does not necessarily alter fungal:bacterial ratios directly, as bacterial biomass also tends to decrease with tillage (Strickland and Rousk, 2010), lower levels of soil moisture under conventional tillage do reduce fungal:bacterial ratios (Frey et al., 1999). As well, tillage reduces AMF community diversity, creating lower diversity subsets of no-tillage communities (Verbruggen et al., 2012). Those AMF that remain are *r-*selected, producing more reproductive spores and fewer soil-exploring hyphae than *K*-selected AMF (Verbruggen and Kiers, 2010). The *r*-selected AMF recover quickly from disturbance but are less efficient at delivering resources to crops (Powell et al., 2009; Verbruggen and Kiers, 2010).

Under SFZM, both disturbed and undisturbed regions are directly adjacent to each other (**Figure 1**). The disturbed region exposes labile organic matter and aerates the soil, providing excellent conditions for nutrient turnover immediately after disturbance (Martens, 2001), while the undisturbed region creates a refuge for slower-growing, more sensitive filamentous fungi and hyphae-intensive AMF. From this refuge, these organisms can quickly re-colonize the mixed and aerated disturbed region. The ‘refuge and recolonization’ process may enhance organic matter production and nutrient cycling. Slow-growing *K*-selected fungi contribute to long-term organic matter pools through necromass production and through the formation of protective soil aggregates (Six et al., 2006; Crowther et al., 2015; Ludwig et al., 2015). As primary decomposers of crop residues, they also have unique ability to access N-rich soil and C-rich crop residues simultaneously, transporting C from residue to soil, and N from soil to residue (Hendrix et al., 1986; Frey et al., 2000, 2003). Tillage disrupts the hyphal networks of these fungi, thereby limiting the production of these services. However, disturbance does enhance residue-soil contact to speed colonization by decomposers. Therefore, the creation of two functionally distinct, adjacent zones under SFZM – an undisturbed fungal refuge and an area where residue is mixed well with soil – should facilitate decomposition of crop residue and the formation of organic matter.

Such refugia may explain enhanced P delivery to maize (*Zea mays* L.) by AMF in SFZM systems (McGonigle and Miller, 1993). P-limitation is a common problem for cereal production in many temperate growing regions, especially on calcareous, P-fixing soils (Holloway et al., 2001). In such a region, young maize plants were found to accumulate greater quantities of P under SFZM (ridge tillage) than under uniform tillage (chisel plow), which was due to greater mycorrhizal activity in the ridge (McGonigle et al., 1990; McGonigle and Miller, 1993, 1996). Based on more recent studies of mycorrhizal P delivery to a variety of plant species, increased P delivery may result from increases in the abundance of Diversisporaceae (formerly Gigasporaceae). This family of AMF develops more extensive soil hyphae and is more effective at delivering P to host plants than other AMF families (Glomeraceae and Acaulosporaceae; Powell et al., 2009). Tillage strongly hinders Diversisporaceae activity (Verbruggen and Kiers, 2010), but the targeted disturbance of ridge top removal and later reformation (**Figure 1**) likely enables them to persist in ridge tillage systems (Ewing et al., unpublished).

In addition to fungi and bacteria, soil fauna may be better protected in SFZM systems. Soil fauna contribute to important agroecosystem services, including decomposition, nutrient cycling, bioturbation, and pest suppression (Coleman et al., 2004; Birkhofer et al., 2008; Suzuki et al., 2013). For example, soil macrofauna facilitate decomposition by fragmenting and redistributing plant residues in the soil profile (Brussaard et al., 2007; García-Palacios et al., 2013). It is well-established that tillage acts as a strong physical filter on soil faunal communities (Roger-Estrade et al., 2010). In a vegetable production system, the combination of reduced tillage (active turnover zone) and no-tillage (soil building zone) in SFZM strip tillage systems maintained higher earthworm and nematode populations compared to conventional, uniform tillage systems (Overstreet et al., 2010). Furthermore, when strip tillage was combined with strategic management of cover crop residues, predatory mite and collembolan (fungivore) densities and nematode community complexity increased compared to conventionally managed systems (Wang et al., 2011).

#### Nitrogen Cycling

Soil functional zone management systems may also enhance crop N nutrition by promoting greater synchrony between soil N availability and crop N requirements. Crop N demand varies over the growing season, and is greatest for row crops during vegetative growth (Olson and Kurtz, 1982; Robertson, 1997), which generally happens in mid- to late- summer. When fertilizer N is supplied at the time of planting, the resulting asynchrony with crop demand can encourage weed growth, lead to inefficient crop use of fertilizer, and drive N loss from soils via denitrification or leaching (Robertson, 1997; Crews and Peoples, 2005; Shanahan et al., 2008). These problems can be addressed by management that synchronizes N supply with peak crop N demand.

**FIGURE 2:**
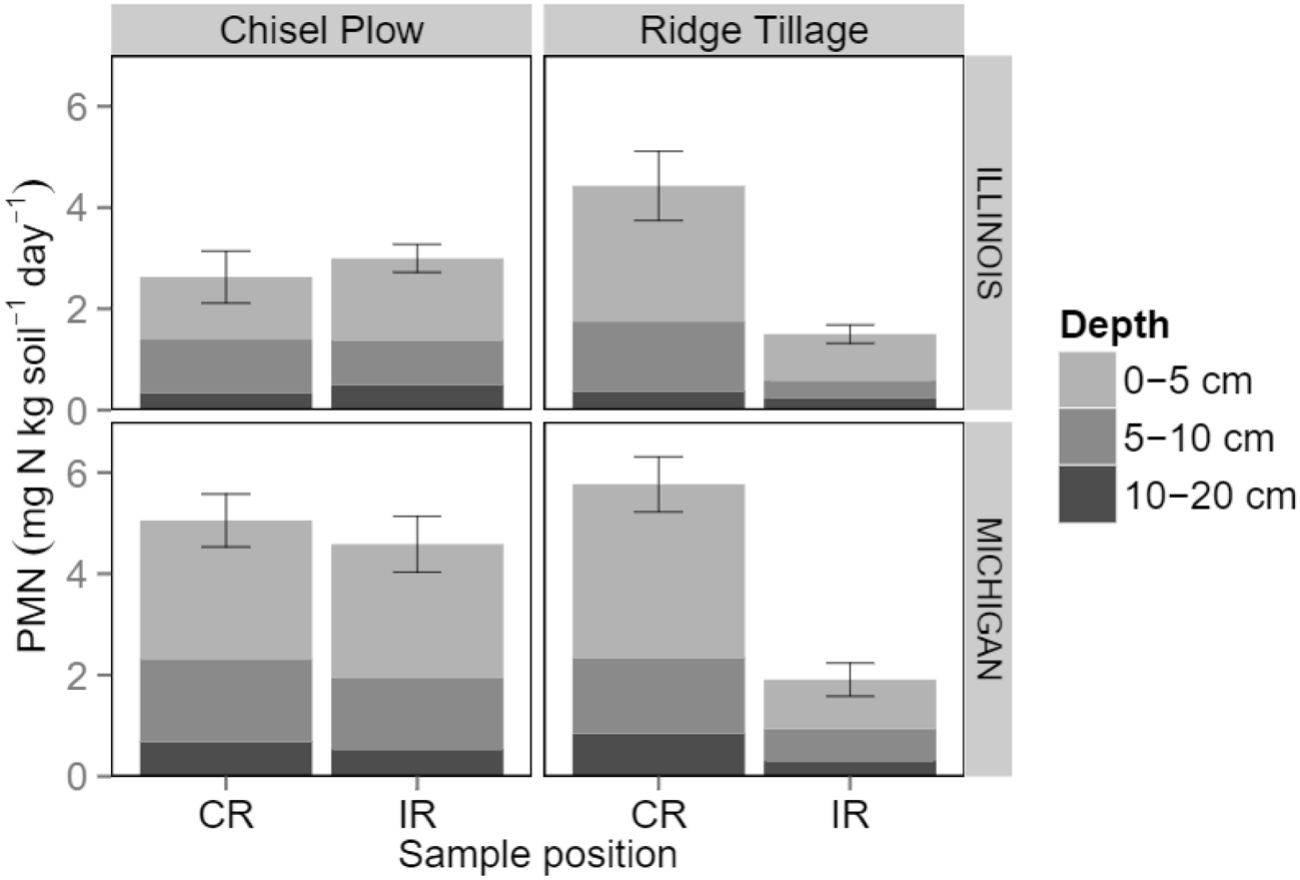
**Potentially mineralizable N (PMN) at different depths and positions (CR: crop row; IR: inter-row) in two maize-soybean cropping systems during mid-summer.** Error bars represent ± 1 SE. Adapted from Kane et al. (2015).

The key to N synchrony may be to manage N supply in both space and time (Shanahan et al., 2008). This is a central feature of SFZM, especially when redistribution of plant residues into the crop row is involved, such as under ridge tillage (John et al., 2004). Under a range of row crops and crop rotations, ridge tillage creates higher concentrations of soil organic C (SOC; Shi et al., 2012), potentially mineralizable N, microbial N (Müller et al., 2009b) and microbial biomass (Bezdicek et al., 2003; Grigera et al., 2007; Müller et al., 2009b) on the ridge-tops of crop rows compared with inter-rows. This spatial concentration of resources and microbial biomass leads to increased microbial activity in the crop row (Clay et al., 1995; Liebig et al., 1995; Müller et al., 2009a), and increases rates of N mineralization (**Figure 2**; Kane et al., 2015). Thus, ridge tillage appears to synchronize potentially mineralizable N supply with crop demand in both space and time, resulting in greater crop N uptake (Gordon et al., 1993; Kane et al., 2015). Similar increases in N mineralization have been observed in the inter-row spaces of strip tillage systems of both maize and orange trees (*Citrus sinensis* L.) Osbeck; Johnstone et al., 2009; Balota and Auler, 2011), but strip tillage was not found to improve N synchrony in a cabbage (*Brassica oleracea* L.) system (Haramoto and Brainard, 2012). This may indicate that the redistribution of plant and soil residues that occurs during ridge tillage is the key to unlocking the N synchrony potential of SFZM. Furthermore, to the extent that SFZM encourages nutrient recycling ecosystem services, then synchronized N can be supplied from internal sources (crop residue, cover crop, or weed residues), reducing the need for fertilizer inputs.

#### Potential Trade-Offs

Despite the wide range of benefits that may result from SFZM, undesirable effects may also arise, creating trade-offs associated with SFZM. Undesirable effects include the potential for increased populations of some pests due to less frequent and less intense tillage operations (Chaplin-Kramer et al., 2011). For example, incidence of Rhizoctonia root rot and parasitic nemadotes increased in no-tillage systems with residue retention compared with conventional tillage (Schroeder and Paulitz, 2006; Govaerts et al., 2007). However, when used in combination with other pest management practices, like diverse crop rotations, SFZM strategies that include an intra-seasonal tillage event, such as ridge tillage, can help disrupt pest populations while maintaining natural enemy populations (McKeown et al., 1998). Pruess et al. (1968) observed clustering of western corn rootworm (*Diabrotica virgifera* Le Conte) eggs in furrow positions and delayed larval development following an intra-seasonal ridging event. They suggested the ridging event relocated the previously uniformly dispersed eggs into the furrow while also burying the eggs under surface debris, lowering soil temperatures, and slowing larval development (Pruess et al., 1968). Additional research on the effects of timing of intra-seasonal tillage on pest and natural enemy populations will be necessary to further minimize pest management trade-offs associated with SFZM.

### Regulating Soil Services

#### Soil Structure, Moisture, and Carbon Storage

The accumulation of SOM in agricultural systems has important implications for soil structure development (Bronick and Lal, 2005; Lal, 2009). SOM is a primary building block of aggregates – it serves to bind and stabilize soil micro-aggregates, which in turn coalesce to form macro-aggregates (Tisdall and Oades, 1982; Bronick and Lal, 2005; Lützow et al., 2006; Karami et al., 2012). Soil tillage and residue management affect aggregate development through their collective influence on SOM quality and accrual. Previous studies have found that SFZM systems increase organic matter (OM) in surface soil layers (0–15 cm) relative to conventional tillage (Angers et al., 1995; Unger, 1995). In turn, SFZM systems, much like no-tillage systems, have been found to increase aggregate stability and average size relative to conventional tillage (Kladivko et al., 1986; Mikha and Rice, 2004; Zibilske and Bradford, 2007). The relative improvements to soil structure in these studies were attributed to minimal tillage-induced disturbance to larger, more fragile aggregates.

The physical encapsulation of OM within soil aggregates plays an important role in the accumulation of soil C (Balesdent et al., 2000; Grandy and Robertson, 2007; Plaza et al., 2013). The OM contained within macro-aggregates is labile and particulate in nature, while micro-aggregate C is more stable, having undergone microbial processing (Elliot, 1986; Plaza et al., 2013; Zhang et al., 2013). Macro-aggregates are highly sensitive to management, with their stability depending largely on plant roots, fungal hyphae, tillage intensity, and microbial activity (Six et al., 2000; Rillig and Mummey, 2006; Zhang et al., 2013). In conventional systems, where macro-aggregate structures are regularly broken down, labile forms of C are released from physical protection resulting in rapid SOM depletion (Grandy and Robertson, 2006, 2007; Panettieri et al., 2015). The reduction in soil disturbance under SFZM increases soil aggregate formation, and the process of concentrating crop residues in inter-row positions has been found to increase concentrations of SOM (Unger, 1995).

**FIGURE 3:**
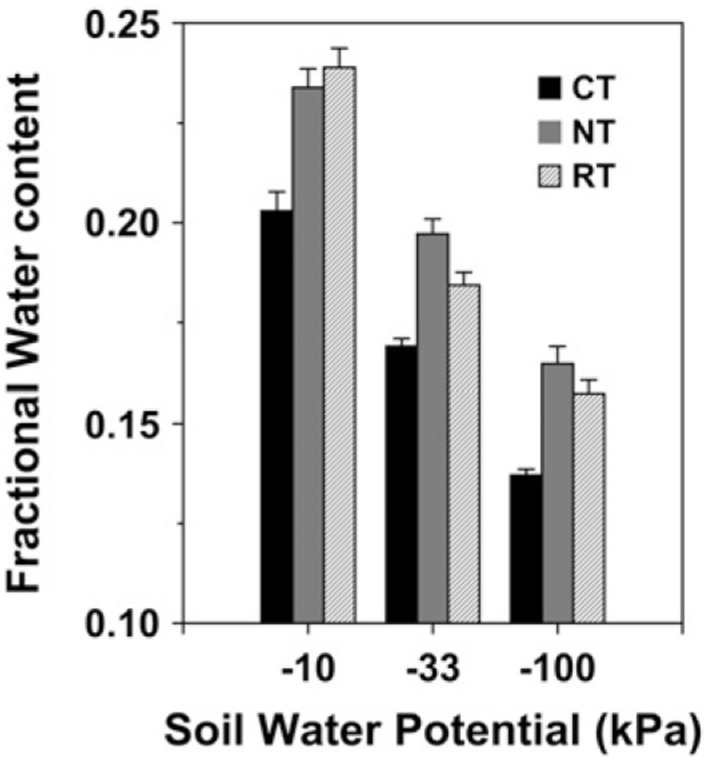
**Water holding capacity at three water potentials in the top 2.5 cm of soil after 13 years of conventional tillage (CT), no-tillage (NT), and ridge tillage (RT).** Error bars represent ± 1 SE. Reproduced from Zibilske and Bradford (2007).

The improvement of soil structure via enhanced aggregate formation under SFZM provides regulating services by facilitating rainfall infiltration and enhancing soil water holding capacity (**Figure 3**; Franzluebbers, 2002; Zibilske and Bradford, 2007). SFZM systems have been shown to conserve soil moisture more effectively than conventional tillage systems (Drury et al., 2006; Zibilske and Bradford, 2007; Williams et al., under review). This feature may be particularly important in terms of adapting agricultural systems to drought stress. Droughts are predicted to increase in frequency and severity with climate change (Gornall et al., 2010; Trenberth et al., 2014). No-tillage has been highlighted as a drought management option due to its ability to conserve soil moisture (Lal, 2004; Powlson et al., 2014). SFZM, because it features zones of no or reduced tillage, may therefore play a crucial role in buffering agricultural systems against drought, while minimizing trade-offs with provisioning services associated with no-tillage (Pittelkow et al., 2015). Put another way, SFZM may help build resilience to climate change while protecting long-term agricultural productivity.

In addition, we hypothesize that the heterogeneous soil environments created by SFZM allow development of greater fungal biomass by providing refugia from tillage disturbance (see Services Produced by Soil Biota above); fungal hyphae play an important role in the formation and stability of soil aggregates (Wilson et al., 2009; Peng et al., 2013; Lehmann and Rillig, 2015). Recent studies lend support to this hypothesis, as reduced tillage systems have been shown to promote greater fungal biomass and diversity relative to conventional tillage systems (van Groenigen et al., 2010; Säle et al., 2015). Furthermore, crops grown under ridge tillage have shown greater mycorrhizal colonization compared with crops grown under uniform tillage systems (McGonigle and Miller, 1993; McGonigle et al., 1999). Thus, by providing greater long-term protection of SOC by enhancing aggregate formation, SFZM could potentially reduce the release of CO_2_ and other greenhouse gasses back to the atmosphere, thereby helping to mitigate the contribution of agriculture to climate change.

#### Additional Regulating Services: The Case of Weed Control

Soil functional zone management may also provide regulating services that contribute to the suppression of weeds. Non-herbicidal weed suppression services will become increasingly valuable as populations of weeds that are resistant to glyphosate and other herbicides continue to become more abundant. The problem of herbicide resistant weeds is especially acute in conventional no-tillage systems, and particularly in those systems that rely on herbicide resistant crops, because of their exclusive reliance on herbicides for weed control (Mortensen et al., 2012). SFZM, through a variety of mechanisms, may reduce weed density and growth, shift the competitive balance from weeds to crops, and provide more opportunities for integrated weed management than conventional no-tillage or other uniformly managed systems.

One way that SFZM can contribute to the management of weeds is through promotion of AMF. AMF can suppress the development of both AMF host and non-host weed species (Jordan et al., 2000; Vatovec et al., 2005), thereby reducing crop yield losses to weeds (Rinaudo et al., 2010; Veiga et al., 2011). Several studies have found negative correlations between AM colonization and crop growth in no-tillage systems relative to conventional tillage, which has been attributed to cooler temperatures in no-tillage crop rows as a result of residue cover (McGonigle and Miller, 1996; McGonigle et al., 1999). SFZM may overcome such drawbacks by removing crop residues from crop rows and concentrating them in relatively undisturbed inter-rows (**Figure 1**). This uncoupling of soil temperatures and residues from areas of soil disturbance allows soil in row positions to warm more rapidly early in spring, while preserving an extensive AMF mycelial network for rapid root colonization in inter-rows (Johnson et al., 1997). Maize grown under ridge tillage has been shown to have increased mycorrhizal colonization and enhanced early season crop performance relative to no-tillage (Vivekanandan and Fixen, 1991; McGonigle and Miller, 1993). When AMF colonize multiple hosts they can increase nutrient transfer to the host that provides the most carbohydrates (Lekberg et al., 2010; Kiers et al., 2011). As such, by improving crop establishment and vigor relative to no-tillage, SFZM can alter interactions between crops and weeds via AMF, improving crop nutrition and performance, and inhibiting weed development. Such improvements have been demonstrated in a strip tillage system, where tomato (*Solanum lycopersicum* L.) performance was improved by AMF when in competition with bahiagrass (*Paspalum notatum* Flügge; Sylvia et al., 2001). However, further research is needed to quantify the contribution of AMF to weed suppression in addition to crop performance within SFZM systems.

Soil functional zone management may enhance weed suppression in other ways, particularly when integrated with cover crops. Cover crops present in inter-rows can suppress weeds through resource and light competition (Liebman and Dyck, 1993; Teasdale, 1996), disruption of weed life cycles (Moyer et al., 2000), physical suppression by cover crop residues (Moore et al., 1994), and release of phytotoxic chemicals (Kruidhof et al., 2009; Teasdale et al., 2012; Samedani et al., 2013). Release of phytotoxic chemicals from cover crop residues can also have negative effects on crop species (Kruidhof et al., 2011; Soltys et al., 2012), and this can be particularly true in uniform tillage systems. SFZM, particularly in ridge tillage systems, removes residues from the crop row and concentrates them in inter-row positions (Hatfield et al., 1998; **Figure 1**). Therefore, by actively managing the placement of phytotoxic cover crop residues, SFZM can minimize some of the potential trade-offs associated with the use of cover crops. The process of concentrating crop residues also promotes survival of soil pathogens in inter-row positions, by increasing inter-row soil moisture content (Cook and Haglund, 1991; Page et al., 2013; Manstretta and Rossi, 2015); weed seeds on or near the soil surface in inter-row positions are then subject to pathogen attack (Caesar, 2005), while crop seeds in the row avoid such attack.

The concentration of crop residues in inter-rows under SFZM may further control weeds by smothering and reducing light penetration to the soil, reducing weed emergence (Forcella and Lindstrom, 1988; Kruidhof et al., 2009). The re-ridging event in ridge tillage, where residues and soil are moved from the inter-row and concentrated on ridges (**Figure 1**), can also serve to smother weeds growing in the crop row (Buhler, 1992). The combination of concentrated crop residues and reduced thermal time accumulation in SFZM systems may provide an additional weed control mechanism.

## SFZM and Provisioning Services

In our presentation of SFZM hitherto, we have sought to establish that improvements in soil regulating and supporting services can be achieved while maintaining existing levels of agricultural output. The successful integration of conventional, intensive agricultural management approaches with more environmentally sustainable practices such as no-tillage would represent a major advance in agronomy. However, given expected increases in global demand for food and other agricultural products by 2050 (Godfray et al., 2010; Tilman et al., 2011), and the need to limit conversion of additional lands to agriculture, it is not sufficient for the world’s existing crop production systems to maintain current levels of production; they must become more productive (Foley et al., 2011; Bommarco et al., 2013; Godfray and Garnett, 2014).

### Temporal Intensification

One way of increasing the productivity of existing agricultural land is through temporal intensification, which aims to expand the annual time period in which harvestable crops are grown. Practices aimed at temporally intensifying agriculture are being increasingly implemented around the world (Ray and Foley, 2013). These include increasing crop harvest frequency per unit area and time by double or triple cropping (Heaton et al., 2013; Ray and Foley, 2013), and earlier planting of cultivars with longer maturation times (Sacks and Kucharik, 2011).

Temporal intensification may improve soil services by reducing or eliminating periods when soil is left bare or fallow. By replacing bare-soil fallows with live plant communities during some or all of the year, temporal intensification can provide a range of soil related regulating and supporting services, such as reduced rates of soil erosion and nutrient leaching (Dabney et al., 2001; Dean and Weil, 2009), increased microbial community size and activity (McDaniel et al., 2014; Tiemann et al., 2015), and weed suppression (Davis and Liebman, 2003; Carrera et al., 2004). In addition, temporal intensification provides opportunities to increase crop rotational diversity (Moore and Karlen, 2013). These factors enhance crop residue, root and exudate production, providing increased C resources for microbial processing (Kong et al., 2011; Tiemann et al., 2015), with subsequent soil quality benefits including long-term C storage and improved soil structure (Grandy and Robertson, 2007; Schmidt et al., 2011; Poeplau and Don, 2015; Tiemann et al., 2015).

Despite the potential benefits of temporal intensification, there are also large potential drawbacks, including reductions in the yields of each crop when multi-cropping is used for temporal intensification (Tonitto et al., 2006; Johnson et al., 2015). Such reductions may be severe if soil resources are exhausted or tied up by previous crops or their residues, or if harvest of one crop delays planting of the next crop. Such delays and the lack of operational flexibility they incur can severely limit production capacity. Other potential drawbacks include damage to soil structure from increases in soil cultivation intensity (Grandy and Robertson, 2006), and greater nutrient leaching and depletion of water resources due to increased fertilization and irrigation (Ju et al., 2009; Ray and Foley, 2013). Soil biodiversity may also be reduced by temporal intensification due to the deleterious effects of increased soil cultivation and elevated input of agrochemicals (Helgason et al., 1998; Mäder et al., 2002). Loss of soil biodiversity may curtail ecosystem functions that generate soil ecosystem services (Bardgett, 2005).

To mitigate these potential downsides while still realizing the inherent benefits of temporal intensification, novel management systems are needed. These systems must enable increases in the amount of product that can be extracted over a given time period while simultaneously protecting soil functional biodiversity and building soil quality. We contend that SFZM is a particularly promising strategy for achieving sustainable temporal intensification because it involves the creation of functionally distinct yet complementary soil zones. Through the integration of conventional, intensive management and reduced tillage practices, these zones are optimized for crop productivity (active turnover zone) and soil protection (soil building zone).

### Dynamics of SFZM: Potential for a Virtuous Cycle Linking Yield and Soil Quality

**FIGURE 4:**
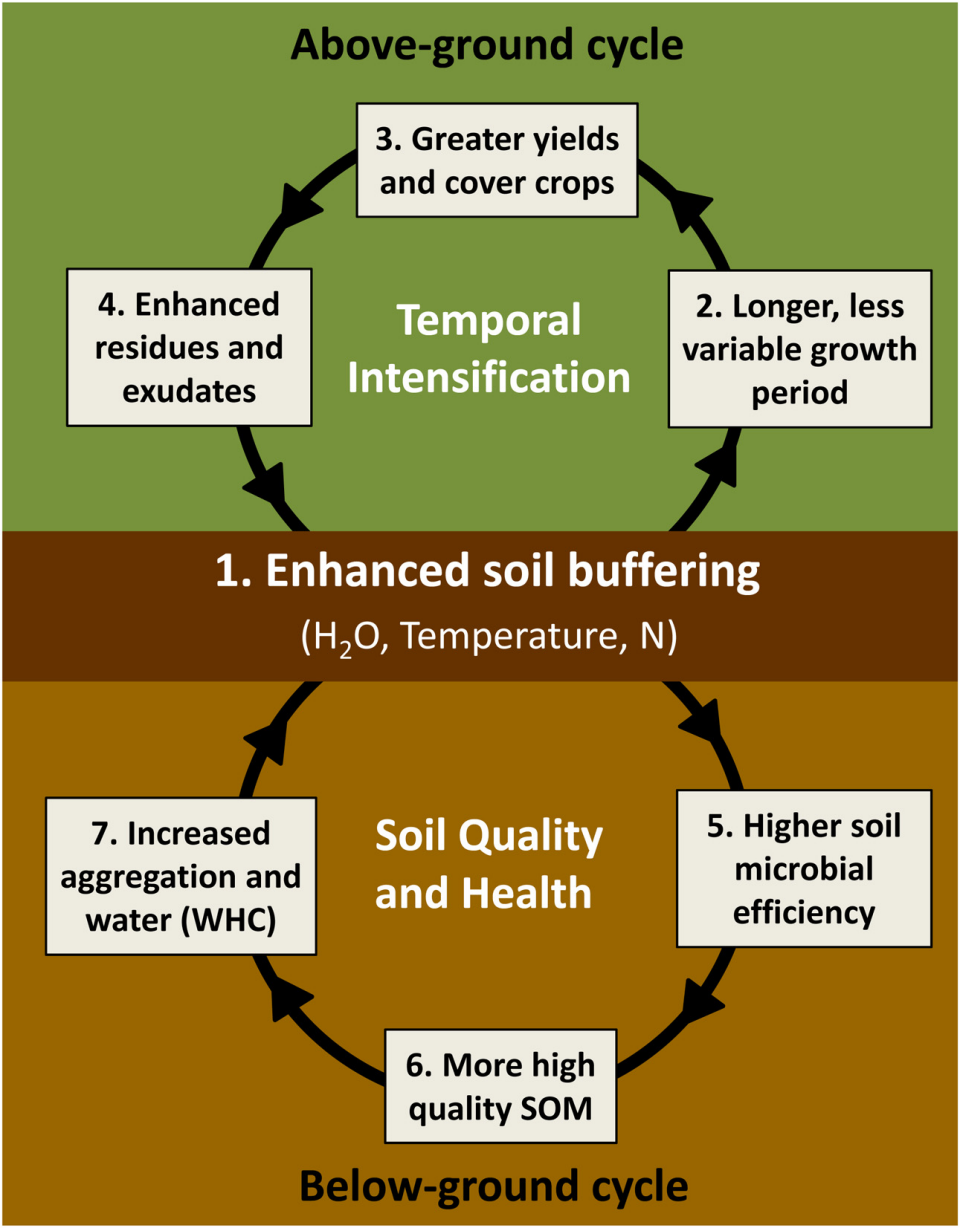
**Proposed ‘virtuous cycles’ of SFZM.** SFZM improves soil hydrothermal and fertility properties (buffering) (1), enabling earlier crop planting and a longer, more stable growth period, even in the face of variable weather patterns (2). This extended growing season supports greater yields from double cropping, crop residue harvest, and more effective cover crop production (3). An extended period of living plant cover enhances crop residue, root, and exudate production (4), resulting in higher soil microbial efficiencies (5) that drive the conversion of residues and microbial biomass into SOM (6). These biologically derived organic matter inputs improve soil quality and health by increasing aggregation, water holding capacity, and plant-available nutrients (7), which together confer and reinforce the soil’s capacity to buffer against variability in rainfall and temperature (1).

We base our hypothesis of joint enhancement in provisioning and other ecosystem services via SFZM on a virtuous cycle model that links above-ground and below-ground processes (**Figure 4**). Specifically, we propose that SFZM engenders a self-reinforcing feedback process that couples improvements in soil regulating and supporting services (below-ground cycle) with improvements in provisioning services via increased field working days (above-ground cycle).

#### Above-Ground Processes in the Virtuous Cycle

A key component of sustainable temporal intensification is increasing the period of time during which crops can be grown and harvested on existing agricultural land. In real terms, this translates into a need for increased field working days, which can be achieved by enabling earlier soil cultivation and planting, by supporting crop growth later in the season, or by a combination of both.

Existing SFZM systems (e.g., ridge and strip tillage), which remove crop residues from crop row positions prior to planting, have been demonstrated to produce seedbed environments that warm and dry rapidly in early spring (Hatfield et al., 1998; Licht and Al-Kaisi, 2005). These seedbeds have similar hydrothermal properties to conventional tillage systems, which in turn have improved hydrothermal properties relative to no-tillage systems, i.e., are warmer and drier, resulting in improved seedling emergence relative to no-tillage (Cox et al., 1990; Kovar et al., 1992; Dwyer et al., 2000). Planting date has a large influence on crop productivity, and delays in planting due to climate fluctuations can severely reduce yields (Deryng et al., 2014). On poorly drained, finely textured soils, or during periods of excessive rainfall, ridge tillage can also improve seedbed hydrothermal conditions above that of conventional tillage, leading to earlier planting, greater accumulation of thermal time and improved yields (Cox et al., 1990; Eckert, 1990; Fausey, 1990). This provides the basis for an important premise of the virtuous cycle model (**Figure 4**): that SFZM increases field working days by allowing cultivation and planting to occur earlier in the season compared to when these operations could occur, for example, in an adjacent field managed with no-tillage approaches. SFZM would also likely outperform conventional tillage in terms of field working days in poorly drained soils or in years with wet springs (**Figure 4**, points 1 and 2).

Soil functional zone management can also extend the growing season by continuing to support crop growth later in the season. Existing SFZM systems concentrate soil moisture into crop inter-row positions (Müller et al., 2009b; Shi et al., 2012), substantially increasing soil moisture above that of conventional systems and maintaining it at levels similar or equivalent to no-tillage (Drury et al., 2003, 2006). These moisture-rich inter-rows may provide an important water resource during critical periods of crop development (Alvarez and Steinbach, 2009). Thus, by altering soil hydrothermal properties, SFZM can increase field working days at both ends of the growing season; allowing soil to be cultivated and/or planted earlier in the season, and maintaining soil moisture in inter-rows that can sustain crop growth later in the season or support planting of winter double crops. In other words, SFZM creates functionally distinct zones that together provide greater soil buffering to climate variability; SFZM buffers against extremes in soil temperature and moisture, and thereby provides a longer, less variable growth period (**Figure 4**, points 1 and 2).

The extension of the growing season afforded by SFZM enables greater utilization of solar radiation both at the beginning and end of the growing season, particularly in northern temperate regions. Longer seasons also allow greater capture of light energy and accumulation of hydrothermal time for both summer and winter crops in double cropping systems, increasing yield potential (Chen et al., 2011; **Figure 4**, point 3). The conservation of soil moisture through late summer in SFZM would also provide a water resource for the establishment of winter crops in double cropping systems, which are currently hampered by growing season duration. By extending the growing season, SFZM has the potential to reduce risks of seasonal crop yield reductions due to delayed harvest under temporal intensification. In addition, the ability of SFZM to enhance soil water conservation could potentially reduce requirements for additional irrigation, as required in some temporally intensified systems (Ray and Foley, 2013).

Temporal intensification may itself also help agriculture become more resilient to climate change. For example, double cropping, facilitated by SFZM, may shift phenologies of some crops, enabling them to avoid peak summer temperatures during critical development phases, when excessive heat can cause severe yield reductions (Seifert and Lobell, 2015). Moreover, SFZM may be particularly suited to support the production-enhancing aspect of temporal intensification because of new technologies for utilizing agricultural biomass from crop residues, and winter-annual cover crops. In the past, biomass crops and crop residues did not contribute to the food supply; however, a variety of new technologies now enable conversion of this biomass into a wide range of foodstuffs for direct and indirect human consumption, as well as biomass feedstocks for bioenergy and bioproducts (Chen and Zhang, 2015). In addition, by enhancing prospects for temporal intensification, SFZM may help reduce the conflict between food and biofuel production by enabling double cropping, potentially supplying both biofuels and food from the same field in the same season (Dale et al., 2010; **Figure 4**, point 3).

#### Below-Ground Processes in the Virtuous Cycle

By enabling an extension to the period of living plant cover, SFZM can also promote increases in the production of root exudates and crop residues (**Figure 4**, point 4). At the most basic level, the production of microbial biomass is governed largely by input quality and microbial physiological traits, such as microbial C-use efficiency (Sinsabaugh et al., 2013; Wieder et al., 2014, 2015). Root exudates and plant residues are primary sources of these C inputs, and drive microbial activity, biomass and community composition (Rasse et al., 2005; Hartmann et al., 2009; Rousk and Frey, 2015). Root exudates, in particular, are highly labile, and contain more reduced C compounds and lower C:N ratios, encouraging higher microbial C-use efficiency (Manzoni et al., 2012). Microbial activity is reduced by periods of sustained soil moisture deficiency (Borken and Matzner, 2009), causing reductions in soil nutrient availability (Emmett et al., 2004; Larsen et al., 2011). In addition, repeated wet-dry cycling leads to pulses of soil C and N mineralization, potentially accelerating SOM mineralization over time (Borken and Matzner, 2009). This diminishes soil water holding capacity and increases susceptibility to future soil moisture deficits. Thus, management that produces improved conditions for microbial growth (e.g., adequate water and temperature, plus greater quantities of root exudates), as can be achieved by SFZM, may sustain greater microbial activity and efficiency, thereby enhancing nutrient turnover processes (**Figure 4**, point 5).

Traditional soil models suggest that it is not possible to maintain soil quality under conditions of intensifying production and greater extraction of soil resources, because removal of crop residues and intensification of tillage and fertilization will deplete SOM (Janzen, 2006; Grandy and Robertson, 2007). This may not be the case in agroecosystems managed to create distinct soil functional zones. Existing SFZM systems, such as ridge tillage, have been found to be similar to no-tillage systems in that they support greater microbial biomass than conventionally tilled systems (Angers et al., 1992; Müller et al., 2009b; Zhang et al., 2013). Emerging experimental and theoretical evidence shows that dead microbial biomass (i.e., necromass) is a significant fraction of SOM (Grandy and Neff, 2008; Schmidt et al., 2011; Cotrufo et al., 2013; Frey et al., 2013; Wieder et al., 2014). The continuous and rapid turnover of living microbial biomass can produce, over time, a considerable amount of necromass (Liang and Balser, 2011), which stabilizes SOM (Simpson et al., 2007; Schmidt et al., 2011; Miltner et al., 2012; Cotrufo et al., 2013; Gleixner, 2013; **Figure 4**, point 6).

Although microbial biomass can be rapidly mineralized by soil organisms due to its favorable energy yield and low C:N ratio (Blagodatskaya et al., 2014), microbial necromass and other microbial by-products can also be selectively preserved via interactions with soil minerals and incorporation into soil aggregates (Lützow et al., 2006; Throckmorton et al., 2015; **Figure 4**, point 7). In fact, microbial necromass, metabolites, and decomposition products account for the majority of stabilized SOM (Simpson et al., 2007; Grandy and Neff, 2008; Kleber and Johnson, 2010; Schmidt et al., 2011). The accumulation of stabilized SOM within soil aggregates in turn improves infiltration of precipitation and increases soil water holding capacity (Franzluebbers, 2002; Zibilske and Bradford, 2007; **Figure 4**, point 7). By encouraging the development of greater microbial biomass, SFZM may halt declines of SOM observed under conventional tillage, and instead contribute positively to SOM accumulation and soil structure development while simultaneously supporting greater yield extraction through temporal intensification.

## Concluding Remarks

Development and implementation of novel agroecological management systems that allow increases in provisioning services (yield) while simultaneously enhancing regulating and supporting ecosystem services are urgently needed. As our review shows, SFZM offers a strategy for integrating the production benefits associated with intensively tilled field crop production systems with the soil ecosystem service benefits associated with no-tillage. In short, SFZM offers the potential to achieve the best of both approaches. The soil heterogeneity produced by SFZM enhances soil functional biodiversity, and allows farmers to harness this biodiversity to elicit desirable ecosystem functions at appropriate times and places. This can lead to greater resource-use efficiency and closer synchrony between soil processes and crop physiological demands. Moreover, the ability of SFZM to favorably alter soil hydrothermal properties allows extension of the growing season, both at the beginning and end. This opens opportunities for increasing agricultural production via temporal intensification. Coupled with improvements to soil regulating and supporting services, SFZM therefore offers a vehicle for optimizing multiple ecosystem goods and services in agricultural systems.

Widespread adoption and refinement of SFZM depends on progress on several fronts. Further research on all aspects of SFZM systems will be required to ensure that service delivery can be optimized to meet specific needs of farmers and society in particular cropping systems and geographies. As well, progress on adoption and refinement of SFZM systems is likely to be strongly affected by societal demand for the full range of regulating and supporting ecosystem services that such systems may be able to provide (Mitchell et al., 2016). The case of ridge tillage in maize-soybean production in central North America is instructive: despite its economic viability (Archer et al., 2002), this form of SFZM is not widely used in the US. In this region, it appears that the perceived value of ecosystem services resulting from ridge tillage do not provide a sufficient incentive for its widespread adoption. However, new incentives are appearing, such as the rapidly growing interest in management systems that promote “soil health” (Lehman et al., 2015), increasing innovation in incentives for agricultural soil C storage (Funk et al., 2015), and more stringent demands for nutrient-use efficiency and other ecosystem services from sustainability-oriented supply chains (Davidson et al., 2014). If there is significant societal demand for the full range of ecosystem services from SFZM, the collective ingenuity of farmers and agricultural engineers can be expected to drive rapid development and implementation of SFZM. This is evidenced by the widespread adoption of zonal tillage techniques in the Central Valley region of California (USA) in response to imperatives to improve resource-use efficiency and environmental performance of production systems in this region (Mitchell et al., 2016).

## Author Contributions

All authors contributed to the manuscript by reviewing literature, discussing and developing ideas, writing text sections and revising drafts of the manuscript. AW, DK, AD, AG, SH, MH, RK, DM, RS, SS, KS, AY, and NJ developed the initial idea for the manuscript and all contributed to framing and general writing (Abstract, Introduction, SFZM, SFZM and ecosystem services, SFZM and provisioning services, Concluding remarks). PE, LA, and AY contributed to SFZM and Regulating soil services. AJ, ML, and YL contributed to Supporting soil services. AW, AD, AG, RS, and NJ contributed to Virtuous cycles. AW and PE developed **Figure 1**; AG and RS developed **Figure 4.**

## Conflict of Interest Statement

The authors declare that the research was conducted in the absence of any commercial or financial relationships that could be construed as a potential conflict of interest.
